# The Evolution and Variety of RFamide-Type Neuropeptides: Insights from Deuterostomian Invertebrates

**DOI:** 10.3389/fendo.2014.00093

**Published:** 2014-06-19

**Authors:** Maurice R. Elphick, Olivier Mirabeau

**Affiliations:** ^1^School of Biological and Chemical Sciences, Queen Mary University of London, London, UK; ^2^Institut Curie, Cancer Genetics Unit, Inserm U830, Paris, France

**Keywords:** RFamide, neuropeptide, receptor, evolution, deuterostome, echinoderm, hemichordate

## Abstract

Five families of neuropeptides that have a C-terminal RFamide motif have been identified in vertebrates: (1) gonadotropin-inhibitory hormone (GnIH), (2) neuropeptide FF (NPFF), (3) pyroglutamylated RFamide peptide (QRFP), (4) prolactin-releasing peptide (PrRP), and (5) Kisspeptin. Experimental demonstration of neuropeptide–receptor pairings combined with comprehensive analysis of genomic and/or transcriptomic sequence data indicate that, with the exception of the deuterostomian PrRP system, the evolutionary origins of these neuropeptides can be traced back to the common ancestor of bilaterians. Here, we review the occurrence of homologs of vertebrate RFamide-type neuropeptides and their receptors in deuterostomian invertebrates – urochordates, cephalochordates, hemichordates, and echinoderms. Extending analysis of the occurrence of the RFamide motif in other bilaterian neuropeptide families reveals RFamide-type peptides that have acquired modified C-terminal characteristics in the vertebrate lineage (e.g., NPY/NPF), neuropeptide families where the RFamide motif is unique to protostomian members (e.g., CCK/sulfakinins), and RFamide-type peptides that have been lost in the vertebrate lineage (e.g., luqins). Furthermore, the RFamide motif is also a feature of neuropeptide families with a more restricted phylogenetic distribution (e.g., the prototypical FMRFamide-related neuropeptides in protostomes). Thus, the RFamide motif is both an ancient and a convergent feature of neuropeptides, with conservation, acquisition, or loss of this motif occurring in different branches of the animal kingdom.

## Introduction

Neuropeptides are evolutionarily ancient mediators of neuronal signaling in nervous systems that have fundamental roles in regulation of physiological processes and animal behavior. They are remarkably diverse, ranging from 3 to >40 residues, but all are derived from larger precursor proteins. Some neuropeptide precursors are simple, giving rise to a single neuropeptide, while others yield multiple related or unrelated neuropeptides (1–3).

A major challenge in neurobiology is to understand the evolutionary and functional significance of neuropeptide diversity in animals. Determining evolutionary relationships between neuropeptides in different phyla has proven to be difficult because they comprise relatively short stretches of amino acids, often with only a few conserved residues. However, recent advances in comparative genomics/transcriptomics are transforming our understanding of neuropeptide signaling systems. Thus, a picture is emerging of a core set of neuropeptide–receptor signaling pathways that can be traced back to the common ancestor of the Bilateria, with neuropeptide orthologs being identified in an increasingly wide range of animal phyla (4–8).

In this review, our attention is focused on neuropeptides that are unified by a common structural characteristic – the presence of a C-terminal Arg–Phe–NH_2_ (RFamide) motif. In vertebrates, five types of RFamides have been identified, which are derived from different precursor proteins: (1) Gonadotropin-inhibitory hormone (GnIH)-type, (2) Neuropeptide FF (NPFF)-type, (3) Pyroglutamated RFamide peptide (QRFP)-type, (4) Prolactin-releasing peptide (PrRP)-type, and (5) Kisspeptin-type (9, 10). Here, we briefly review how these diverse RFamides were discovered and then we proceed to our main objective – to explore the phylogenetic distribution and evolutionary origins of these neuropeptides. Our primary focus will be on deuterostomian invertebrates (urochordates, cephalochordates, hemichordates, and echinoderms) because these animals share a more recent common ancestor with vertebrates than the protostomian invertebrates (e.g., arthropods, nematodes, annelids, and mollusks) (11). However, in reviewing research on RFamides, the story begins (and ends) with what at first sight might be considered an inauspicious protostomian invertebrate – the sunray venus clam *Macrocallista nimbosa*, a bivalve mollusk.

## FMRFamide – The Prototypical RFamide-Type Neuropeptide

The tetrapeptide Phe–Met–Arg–Phe–NH_2_ (FMRFamide) was purified from extracts of ganglia from the clam *Macrocallista nimbosa* on account of its cardioexcitatory activity. A chromatographically distinct component of molluscan ganglia that stimulates beating of quiescent molluscan hearts, known as peak C, was first reported in 1967 (12). Ten years later Price and Greenberg identified peak C as FMRFamide, reporting their finding in the journal *Science* (13). FMRFamide might have remained an obscurity of molluscan pharmacology were it not for the discovery that FMRFamide-like peptides occur throughout the animal kingdom. For example, using antibodies to FMRFamide, cross-reacting peptides were detected immunocytochemically in the nervous systems of an insect, a fish, and a mammal (14). And so began an era in which neuropeptides that share a C-terminal RFamide motif with FMRFamide were identified in a wide range of taxa, including cnidarians, nematodes, insects, and vertebrates (15, 16). A comprehensive survey of the diverse set of RFamide-type neuropeptides that have been identified in the animal kingdom is beyond the scope of this review, where the focus is on RFamides that were first discovered in vertebrates. However, we will take on a broader phylogenetic perspective in the last section of this review, highlighting, for example, RFamide-type neuropeptides that have ancient bilaterian origins but which have been lost in the vertebrate lineage.

## From LPLRFamide to GnIH: The Bird Brain’s Contribution to RFamide Discovery in Vertebrates

The first RFamide-type neuropeptide to be identified in a vertebrate was purified from chicken brain extracts on account of its cross-reactivity with antibodies to FMRFamide. Reporting in the journal *Nature* in 1983, the purified peptide was identified as the pentapeptide Leu–Pro–Leu–Arg–Phe–NH_2_ (LPLRFamide) (17). Seventeen years later, using antibodies to RFamide, an RFamide-type neuropeptide was purified from quail brain extracts and identified as the dodecapeptide SIKPSAYLPLRFamide (18). Therefore, it is likely that the LPLRFamide peptide originally isolated from chicken brain was a fragment of a homolog of this dodecapeptide. Investigation of the physiological roles of SIKPSAYLPLRFamide has revealed that it inhibits pituitary release of gonadotropic hormones – hence this peptide was named GnIH. The GnIH precursor contains two related peptides known as GnIH-RP1 and GnIH-RP2, which have the C-terminal motif LPxRFamide (where x is L or Q) (19). Importantly, GnIH-like neuropeptides that suppress reproductive activity have also been identified in mammals (20, 21). Furthermore, the orphan receptor GPR147 has been identified as the G-protein coupled receptor that mediates effects of GnIH-type neuropeptides. GPR147 is also now known as NPFFR1 or NPFF1 (22–24).

Analysis of the phylogenetic distribution of GnIH-type neuropeptides has revealed that they occur throughout the vertebrates, from primitive agnathan vertebrates through bony fish and amphibians to reptiles, birds, and mammals (25, 26). A comprehensive survey of the properties and functions of GnIH-type neuropeptides in each of the vertebrate classes is beyond the scope of this review and has been discussed elsewhere. Therefore, we will focus here on studies in the most primitive extant vertebrates, the agnathans (lamprey, hagfish). A cDNA encoding a precursor of two GnIH-type peptides has been identified in the sea lamprey *Petromyzon marinus*, with expression revealed in the hypothalamus and gonads (26). Interestingly, injection of the GnIH-type peptides stimulates expression of GnRH and gonadotropins in lamprey, which contrasts with the inhibitory effects of GnIH in birds and mammals. Thus, the physiological role of GnIH as a regulator of reproductive processes can be traced back to the common ancestor of vertebrates, but inhibitory or stimulatory effects are observed in different vertebrate lineages (26).

## F8Fa (NPFF) and A18Fa (NPAF): Bovine FMRFamide-Like Immunoreactive Peptides and Prototypes for the Neuropeptide FF Family

The first RFamides to be identified in mammals were purified from extracts of bovine brain, employing antibodies to FMRFamide in a radioimmunoassay. Two neuropeptides were identified: FLFQPQRFamide (F8Fa or NPFF) and AGEGLSSPFWSLAAPQRFamide (A18Fa or NPAF), which have a common C-terminal motif – PQRFamide (27). Subsequently, it has been found that these peptides are derived from the same precursor protein (28, 29) and exert effects by binding to the G-protein coupled receptor GPR74, which is also referred to as NPFFR2 or NPFF2 (22, 24, 30).

Early on it was found that NPFF and NPAF attenuate morphine-induced antinociception and cause hyperalgesia (27); consistent with these effects, NPFF2 receptors are expressed in the dorsal horn of the spinal cord (31). However, evidence of roles in regulation of other physiological processes has been obtained subsequently; for example, increasing blood pressure and slowing heart rate (32–34).

Analysis of the phylogenetic distribution of NPFF/NPAF-type neuropeptides has revealed that they occur throughout the vertebrates, from primitive agnathan vertebrates through bony fish and amphibians to reptiles, birds, and mammals (35). For example, in the agnathan *P. marinus* (sea lamprey) a cDNA encoding a precursor protein that gives rise to three neuropeptides with a C-terminal PQRFamide motif has been identified (36). The PQRFamide-type precursor is expressed in several regions of the lamprey brain, including the hypothalamus, mesencephalon, and medulla oblongata (36). Interestingly, *in vivo* pharmacological tests have revealed that one of the PQRFamide-type peptides triggers increased expression of GnRH-II in the lamprey (37). In hagfish, there are two PQRFamide-type precursors and *in vitro* tests with one of the derived PQRFamide-type peptides revealed stimulation of gonadotropin expression in the hagfish pituitary (35).

## RFamides as Ligands for Orphan G-Protein Coupled Receptors: Discovery of QRFP, PrRP, and Kisspeptin

By the late 1990s, a growing number of orphan G-protein coupled receptors had been cloned and sequenced on account of their similarity with known receptors. And so began an era in which the search for endogenous ligands for these receptors became a priority and between 1998 and 2003, three novel types of RFamide neuropeptides were discovered in this period of receptor de-orphanization or “reverse pharmacology” (38, 39).

## QRFP

In 2003, the endogenous ligand for the orphan receptor GPR103 was identified as a 26-residue peptide with a C-terminal RFamide motif, which is known pyroglutamylated arginine–phenylalanine-amide peptide (QRFP) or 26RFa (40–42). Subsequent studies have revealed that QRFP is expressed in regions of the hypothalamus involved in control of feeding behavior and accordingly pharmacological studies have revealed that this peptide increases intake of a high fat diet (43–46).

Analysis of the phylogenetic distribution of QRFP-type neuropeptides has revealed that they occur throughout the vertebrates, from bony fish and amphibians to reptiles, birds, and mammals (47, 48). The occurrence of QRFP-type neuropeptides in agnathans has, as yet, not been reported; however, notwithstanding secondary loss, it is expected because the phylogenetic distribution of QRFP-type neuropeptides extends to invertebrates [see Figure 2B; (8)] and because a QRFP-type receptor is present in lamprey (48). Investigation of the physiological roles of the QRFP-type neuropeptide in a teleost species, the goldfish *Carassius auratus*, revealed that injection of synthetic QRFP caused an increase in serum gonadotropins, but only at the highest dose of QRFP tested (1 μg/g body weight). Interestingly, upregulation of hypothalamic QRFP precursor mRNA expression was observed following 4 days of food deprivation in goldfish, indicating that QRFP-type neuropeptides may have an evolutionarily ancient role in regulation of food intake in vertebrates (47). Further studies are now needed to investigate more widely the physiological roles of QRFP-type neuropeptides in non-mammalian vertebrates.

## PrRP

In 1998, endogenous ligands for the orphan receptor GPR10 were isolated from hypothalamic extracts and identified as a 31-residue peptide with a C-terminal RFamide motif and a N-terminally truncated 20-residue isoform of the 31-residue peptide. Investigation of the physiological roles of these peptides revealed that they stimulate release of prolactin from anterior pituitary cells and hence, they were named PrRPs (49). However, the physiological relevance of this *in vitro* effect of PrRPs has been questioned and alternative roles in regulation of feeding and stress hormone release have been proposed (50, 51).

Analysis of the phylogenetic distribution of PrRP-type neuropeptides has revealed that they occur throughout the vertebrates from primitive agnathans (52) to bony fish and tetrapods [(53); Figure 2C]. Interestingly, phylogenomic analysis has led to the proposal that PrRP and its cognate receptor originated from the neuropeptide Y (NPY) peptide-receptor system following the genome duplications that occurred during the early evolution of vertebrates (53). Thus, the evolution and physiological roles of PrRP-type peptides needs to be considered in the context of a bilaterian family of neuropeptides that includes NPY in vertebrates (C-terminal RYamide motif) and invertebrate neuropeptide F (NPF)-type peptides, which have a C-terminal RFamide motif [(54); see also below for further discussion of this topic].

Comparative analysis of the physiological roles of PrRP-type peptides has revealed that PrRP-type peptides stimulate prolactin release in bony fish (55), indicating that this is an ancient role in vertebrates. Furthermore, evidence of roles for PrRP-type peptides in regulation of feeding behavior and growth hormone release in non-mammalian vertebrates has been obtained (52, 56).

## Kisspeptin

In 2001, the endogenous ligands for the orphan receptor GPR54 were identified as a 54-residue peptide with a C-terminal RFamide motif and N-terminally truncated isoforms of the 54-residue peptide comprising 13 or 14 residues. It was discovered that these peptides are derived from metastasis-suppressor protein KiSS-1 and hence they were named kisspeptins (57). Subsequently, evidence that kisspeptins are important physiological regulators of reproductive development was obtained. Thus, loss-of-function mutations in the kisspeptin receptor (GPR54) cause a failure to progress through puberty due to hypogonadotropic hypogonadism (58).

Analysis of the phylogenetic distribution of kisspeptins has revealed homologs throughout the vertebrates, from agnathans to mammals (59). Furthermore, in some vertebrates up to three genes encoding precursors of kisspeptins have been found; for example, in the coelacanth, a sarcopterygian fish, and the elephant shark, a chondrichthyan fish. Accordingly, multiple copies of candidate kisspeptin receptors are found in some vertebrates; for example, in the coelacanth and the spotted gar (an actinopterygian fish), there are four genes encoding kisspeptin-type receptors and phylogenomic analysis indicates this reflects the two rounds of whole genome duplication that are thought to have occurred in a basal ancestral vertebrate (60). However, in the majority of vertebrates there has been loss of one or more kisspeptin precursor and kisspeptin receptor paralogs. For example, in the zebrafish *Danio rerio* there are two kisspeptin precursors (61), while the most extreme loss is seen in some bird species where no genes encoding a kisspeptin precursor or kisspeptin receptor have been found (62). Investigation of the physiological roles of kisspeptins in non-mammalian vertebrates indicates that the kisspeptin signaling system has an ancient role in regulation of reproductive development (63).

## The Phylogenetic Distribution and Evolutionary Origins of Vertebrate RFamide-Type Neuropeptides: Insights from Deuterostomian Invertebrates

**Figure 1:**
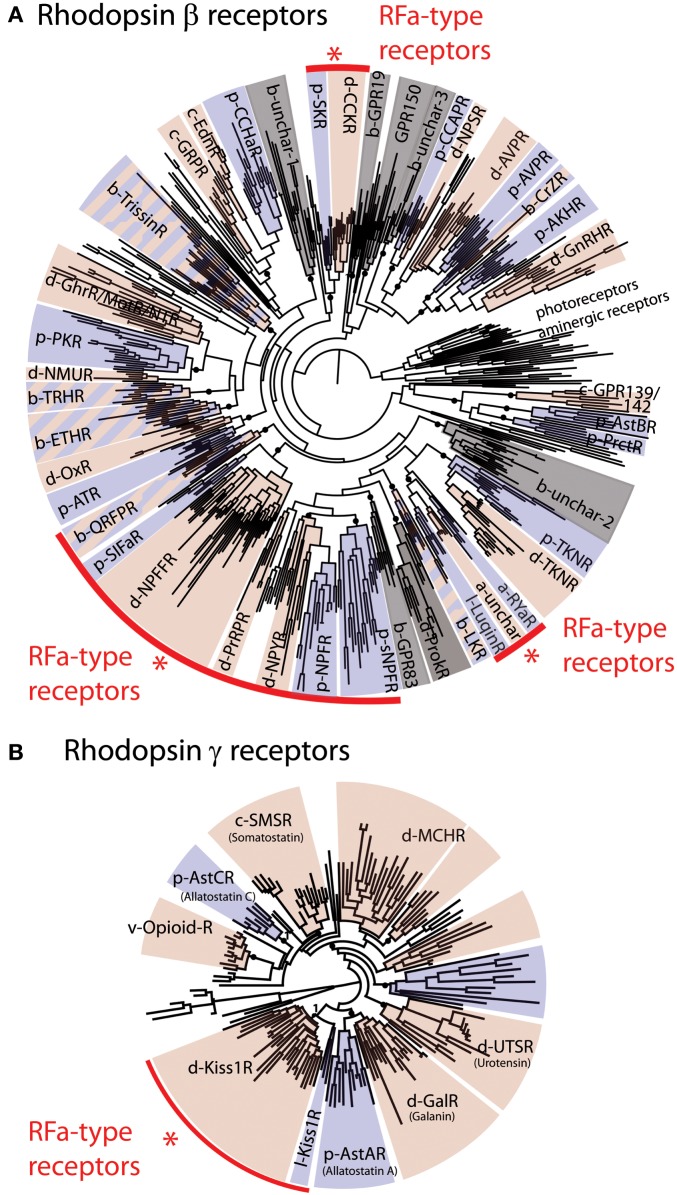
**Phylogenetic analysis of bilaterian rhodopsin β-type (A) and rhodopsin γ-type (B) receptors**. The red arcs highlight four groups that include receptors that are known to be activated by RFamide-type peptides. These include a large group of receptors for QRFP (pyroglutamylated RFamide peptide), SIFa (SIFamide), NPFF (Neuropeptide FF and Gonadotropin-inhibitory hormone), PrRP (Prolactin-releasing peptide), NPY/NPF (Neuropeptide Y/F), sNPF (short Neuropeptide F), and Luqin, and two isolated groups of RFamide-type receptors, CCK/SK (Cholecystokinin/Sulfakinin), and Kiss1 (Kisspeptin) receptors. In **(A)** rhodopsin **α**-type receptors (photoreceptors and aminergic receptors) are included as an outgroup. The prefixes b-, d-, p- designate subgroups of, respectively, bilaterian, deuterostomian, and protostomian receptors. Deuterostomian and protostomian clades have been colored in pink and blue, respectively. Gray sections of the trees correspond to groups of receptors for which either only deuterostomian or protostomian ligands are known. The fact that most of the rhodopsin β-type RFamide receptors fall in the same region of the tree suggests that these probably originated from a common ancestral RFamide-type neuropeptide signaling system. However, the occurrence of other groups of receptors that are activated by RFamides (CCK, Kiss1) indicates that RFamide-type neuropeptides have evolved independently at least three times in bilaterian history. Figure adapted from Ref. (8).

Orthologs of receptors for vertebrate RFamide-type neuropeptides occur in deuterostomian and protostomian invertebrates (8), as illustrated in Figure 1. Thus, based on this phylogenetic distribution, the evolutionary ancestry of NPFF/GnIH-type, QRFP-type, and kisspeptin-type receptors can be traced back to the common ancestor of the bilaterians. The PrRP receptor is an exception in as much as orthologs of this receptor are only found in deuterostomes.

**Figure 2:**
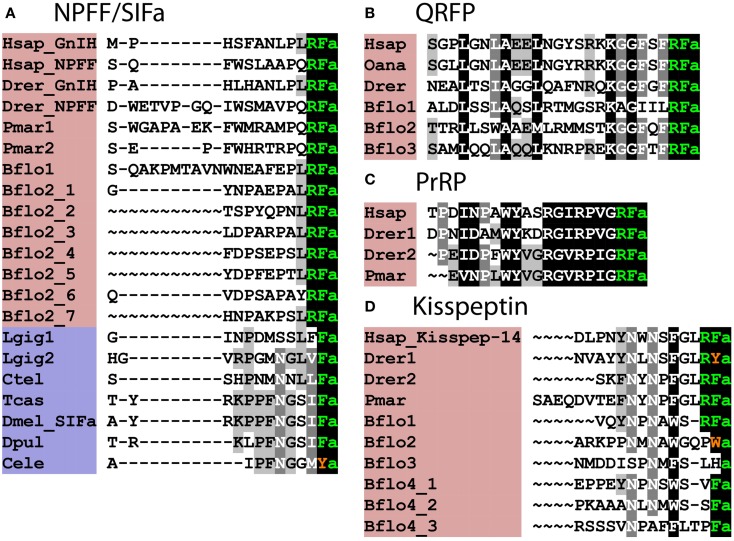
**Alignment of known orthologous peptides from (A) bilaterian NPFF/SIFa (B), chordate QRFP, (C) vertebrate PrRP, and (D) chordate kisspeptin families**. The names of deuterostomian and protostomian peptides are shaded in pink and blue, respectively. The C-terminal amide groups are represented by an “a” at the end of aligned sequences. The lamprey sequences are from Ref. (26), other vertebrate and *B. floridae* sequences are from Ref. (8), the *Lottia gigantea* sequences are from Ref. (4), and the *Capitella teleta* sequence is from Ref. (5). For vertebrate sequences, only one peptide from each precursor is shown in the alignment. Genbank (GI) or JGI IDs of all precursor sequences are listed below: **(A)** alignment of chordate NPFF/SIFa peptides: Hsap_GnIH, *Homo sapiens* GnIH (GI:11125707); Hsap_NPFF, *Homo sapiens* NPFF precursor (GI:219878493); Drer_GnIH, *Danio rerio* GnIH precursor (GI:283825363), *Danio rerio* NPFF precursor (GI:116078056); Pmar1, *Petromyzon marinus* NPFF/GnIH precursor 1 (GI:88595366); Pmar2, *Petromyzon marinus* NPFF/GnIH precursor 2 (GI:374849285); Bflo1, *Branchiostoma floridae* NPFF/GnIH precursor 1 (GI:260808912); Bflo2_1–7, *Branchiostoma floridae* NPFF/GnIH precursor 2 (GI:260829186); Lgig1, *Lottia gigantea* SIFamide precursor 1 (JGI: 176362); *Lottia gigantea* SIFamide precursor 2 (JGI: 175046); Ctel, *Capitella teleta* SIFamide precursor (GI:161198869); Tcas, *Tribolium castaneum* SIFamide precursor (GI:189239683); Dmel_SIFa, *Drosophila melanogaster* SIFamide precursor (GI:386768581); Dpul, *Daphnia pulex* SIFamide precursor (JGI: 260818); Cele, *Caenorhabditis elegans* SIFamide precursor (GI:392894563). **(B)** Alignment of chordate QRFP peptides: Hsap, *Homo sapiens* pyroglutamylated RFamide peptide precursor (GI:38016139); Oana *Ornithorhynchus anatinus* QRFP precursor (GI:620943939); Drer, *Danio rerio* QRFP precursor (GI:528509692); Bflo1–3, *Branchiostoma floridae* QRFP precursors (GI:260828082, GI:260828080, and JGI:107075). **(C)** Alignment of vertebrate PrRP peptides: Hsap, *Homo sapiens* prolactin-releasing peptide/hormone (GI:7705679); Drer1-2, *Danio rerio* prolactin-releasing peptide precursors (GI:350539516, GI:528519441); Pmar, *Petromyzon marinus* prolactin-releasing peptide precursor (Ensembl scaffold: GL490889). **(D)** Alignment of Kisspeptins: Hsap_Kisspep-14, *Homo sapiens* KiSS-1 metastasis-suppressor precursor (GI:116829963); Drer1-2, *Danio rerio* KiSS-1 precursors (GI:157061759, GI:217272819); Pmar, *Petromyzon marinus* KiSS-1 precursors (GENSCAN00000116455); Bflo1-4, *Branchiostoma floridae* KiSS-1 precursors (GI:260826607, GI:260793233, GI:260826605, and GI:260827077).

The discovery of invertebrate orthologs of vertebrate receptors for RFamide-type neuropeptides is fascinating because it provides a basis for discovery of ligands for these receptors and investigation of their physiological roles in invertebrates. An example of where this has been accomplished is the discovery that SIFamide-type neuropeptides are ligands for protostomian orthologs of NPFF/GnIH-type receptors (64). SIFamides share limited sequence similarity with vertebrate NPFF/GnIH-type neuropeptides (Figure 2A); however, analysis of the physiological roles of SIFamide in *Drosophila* indicates that it acts to suppress reproductive behavior (65). This is intriguing because it is consistent with the role of GnIH as an inhibitory regulator of reproductive processes in vertebrates (20). Thus, it appears that the evolutionary origin of GnIH/SIFamide-type neuropeptides as reproductive inhibitors may trace back to the common ancestor of the Bilateria (66).

Little is known about the molecular identity of peptide ligands for invertebrate orthologs of other vertebrate RFamide-type receptors. Therefore, here we have addressed this issue, focusing on deuterostomian invertebrates. These include two sub-phyla of the phylum Chordata – the urochordates [e.g., the sea squirt *Ciona intestinalis*; (67)] and the cephalochordates [e.g., *Branchiostoma floridae*; (68)]. Other deuterostomian phyla are the hemichordates [e.g., acorn worm *Saccoglossus kowalevskii*; (69)] and the echinoderms [e.g., the sea urchin *Strongylocentrotus purpuratus* (70)], which are sister phyla in a clade of deuterostomian invertebrates known as the ambulacraria (11).

## GnIH/NPFF-Type Neuropeptides in Deuterostomian Invertebrates

Orthologs of GnIH/NPFF-type receptors are not present in the sea squirt *C. intestinalis* and therefore, it appears that this neuropeptidergic system has been lost in the urochordate lineage. In the cephalochordate *B. floridae*, there is an expanded family of GnIH/NPFF-type receptors (8) and accordingly at least two precursors of putative peptide ligands for these receptors have been identified, with the predicted neuropeptide products sharing a common C-terminal PxRFamide motif (where *x* is variable) with vertebrate GnIH/NPFF-type neuropeptides [(8); Figure 2A].

GnIH/NPFF-type neuropeptides in vertebrates can be divided into two functional groups – peptides that are PQRFamides, which modulate nociception (28) and LRFamides, which inhibit the release of gonadotropins (19). Peptides derived from both of the GnIH/NPFF-type precursors in *B. floridae* are LRFamides, suggesting that the ancestral GnIH/NPFFamide-type neuropeptides in chordates were LRFamides that may have had a role in regulation of reproduction. Furthermore, the LxFamide motif is conserved in lophotrochozoan (e.g., *Capitella* and *Lottia*) SIFamide-type peptides, suggesting that a leucine at position-2 relative to a C-terminal amidated phenylalanine and a role in reproduction are ancient characteristics of this family of orthologous peptides.

As highlighted above, SIFamide-type neuropeptides have been identified as ligands for GnIH/NPFF-type receptors in protostomian invertebrates. However, SIFamides share very limited sequence similarity with chordate GnIH/NPFF-type peptides; in fact the only universally shared feature is a C-terminal Phe–NH_2_ motif (Figure 2A), although this does extend to Leu–x–Phe–NH_2_ in some cases. Thus, when comparing chordate GnIH/NPFF-type peptides and protostomian SIFamides, sequence divergence has rendered the orthologous relationship between these peptides almost completely unrecognizable. In this context, it would be interesting to identify putative ligands for GnIH/NPFF/SIFamide-type receptors in non-chordate deuterostomes.

Recently, it was proposed that SALMFamide-type neuropeptides may be echinoderm homologs of GnIH/NPFF/SIFamide-type neuropeptides (66). The rationale for this hypothesis was that L-type SALMFamides share sequence similarity with some SIFamides – in particular, the C-terminal SxLxFamide motif. However, we have not obtained support for this hypothesis from analysis of the occurrence of GnIH/NPFF/SIFamide-type receptors in echinoderms. Thus, orthologs of GnIH/NPFF/SIFamide-type receptors do not appear to be present in the sea urchin *S. purpuratus* (8). Therefore, the sequence similarity shared by SALMFamides and some SIFamides may reflect convergence and it appears, based on the data currently available, that GnIH/NPFF/SIFamide-type neuropeptide signaling may have been lost in the echinoderm lineage.

The hemichordates are a sister phylum to the echinoderms and a GnIH/NPFF-type receptor has been identified in the acorn worm *S. kowalevskii* (8). Therefore, the presence of a gene encoding a GnIH/NPFF-type neuropeptide precursor is anticipated, but it remains to be discovered. If the endogenous ligand for the GnIH/NPFF/SIFamide-type receptor in *S. kowalevskii* is identified, it would be fascinating to investigate the physiological roles of this peptide. Does it, for example, act as an inhibitor of reproductive processes in *S. kowalevskii*? – a role that would be consistent with the physiological roles of GnIH and SIFamide in vertebrates and *Drosophila*, respectively.

## QRFP-Type Neuropeptides in Deuterostomian Invertebrates

Orthologs of vertebrate QRFP-type receptors are present in the cephalochordate *B. floridae*, the hemichordate *S. kowalevskii*, the echinoderm *S. purpuratus*, and in lophotrochozoan protostomes but not in urochordates or ecdysozoan protostomes (8). Thus, the evolutionary origin of the QRFP-type peptide signaling system can be traced back to the common ancestor of the Bilateria but with subsequent loss in ecdysozoan protostomes and urochordates.

Three precursors of candidate ligands for QRFP-type receptor(s) have been identified in *B. floridae*. Like most QRFP-type neuropeptides in vertebrates, all three peptides are predicted to comprise 25 residues with a C-terminal RFamide motif. Other features in common with vertebrate QRFP-type neuropeptides include a conserved leucine residue at position four, a conserved alanine residue at position eight, a conserved lysine residue at position 18, and a conserved glycine residue at position 20 [(8); Figure 2B].

QRFP-type receptors are present in echinoderms (*S. purpuratus*) and hemichordates (*S. kowalevskii*) (8); Table S1 in Supplementary Material) but precursors of QRFP-type peptides have as yet not been identified in these phyla. It has been noted though that F-type SALMFamide neuropeptides in echinoderms are similar to vertebrate QRFP-type peptides in having a C-terminal FxFamide motif (where x is variable) (71). However, the organization of F-type SALMFamide precursors is different from that of QRFP-type precursors because they comprise multiple copies of shorter peptides, ranging in length from seven to twenty residues (72). This contrasts with QRFP-type precursors that have been identified in chordates, which comprise a single copy of a 25-residue peptide (Figure 2B). More extensive analysis of echinoderm and hemichordate genome/transcriptome sequence data is now required to investigate the existence of precursors of QRFP-type peptides in these non-chordate deuterostomian invertebrates. Likewise, QRFP-type receptors are present in lophotrochozoan protostomes (8) but candidate peptide ligands for these receptors have yet to be identified. If this can be accomplished, then comparative functional analysis of QRFP-type peptides in a variety of invertebrates would provide fascinating insights into the origins and evolution of the physiological roles of this ancient bilaterian neuropeptidergic signaling system.

## PrRP-Type Neuropeptides in Deuterostomian Invertebrates

Orthologs of vertebrate PrRP-type receptors are present in the cephalochordate *B. floridae* and the hemichordate *S. kowalevskii* but not in urochordates, echinoderms, or protostomes (8). We have identified one and three PrRP-type receptors in the hemichordate *S. kowalevskii* and the cephalochordate *B. floridae*, respectively, but analysis of genome/transcriptome data for these species has thus far not yielded candidate precursors of peptide ligands for these receptors [(8); Table S1 in Supplementary Material]. Nevertheless, the existence of PrRP-type receptors in *B. floridae* and *S. kowalevskii* indicates that the duplication of a NPY-type receptor gene and a NPY-type precursor gene that is proposed to have given rise to the PrRP peptide-receptor system occurred earlier in animal evolution than has been proposed previously (53). Our data indicate that these gene duplications occurred in a common ancestor of deuterostomes, with subsequent loss in urochordates and echinoderms.

## Kisspeptin-Type Neuropeptides in Deuterostomian Invertebrates

Orthologs of vertebrate kisspeptin-type receptors are present in the cephalochordate *B. floridae*, the hemichordate *S. kowalevskii*, the echinoderm *S. purpuratus*, and in lophotrochozoan protostomes but not in urochordates or ecdysozoan protostomes [(8); Table S1 in Supplementary Material]. Based on these findings, the evolutionary origin of kisspeptin-type peptide signaling can be traced back to a common ancestor of the Bilateria but with subsequent loss in urochordates and ecdysozoan protostomes.

In *B. floridae*, an expanded family of 16 kisspeptin-type receptors and 4 precursors of kisspeptin-like peptides have been identified [(8); Figure 2D; Table S1 in Supplementary Material]. One of the precursors (Bflo1; GI:260826607) contains a peptide with a putative C-terminal RFamide motif, in common with vertebrate kisspeptins (Figure 2D). Another kisspeptin-type precursor (Bflo4; GI:260827077) gives rise to peptides with putative C-terminal VFamide, SFamide, and PFamide motifs and two paralogous precursors (Bflo2; GI:260793233 and Bflo3; GI:260826605) yield peptides with a C-terminal Wamide and Hamide, respectively. However, features that are conserved between all chordate kisspeptins are two conserved asparagine residues located seven or eight, and five or six residues from the C-terminal amidated residue (Figure 2D) and a conserved phenylalanine or tryptophan located three or four residues from the C-terminal amidated residue (Figure 2D).

Hitherto, kisspeptin-type peptides have only been identified in chordates (8). However, the occurrence of kisspeptin-type receptors in *S. kowalevskii* and *S. purpuratus* (8) indicates that kisspeptin-type peptides may exist in hemichordates and echinoderms. Likewise, the presence of kisspeptin-type receptors in lophotrochozoan invertebrates indicates that kisspeptin-type peptides may exist in these animals, but they remain to be discovered. If kisspeptin-type peptides can be identified in ambulacrarians (hemichordates and echinoderms) and lophotrochozoans (e.g., mollusks and annelids) then investigation of their functions in these animals may provide insights into the ancestral physiological roles of kisspeptins in the common ancestor of bilaterians.

## Other RFamide-Type Neuropeptides in Bilaterians

The primary focus of this review has been on neuropeptide families where vertebrate representatives have a C-terminal RFamide motif. However, there are also other bilaterian neuropeptide families that include RFamide-type peptides but where vertebrate representatives have either lost this feature or have been lost altogether. Furthermore, some RFamide-type neuropeptides appear to have evolved only within the protostomian lineage. Below, we briefly discuss some examples and in so doing illustrate that in characterizing neuropeptides as “RFamides,” the representatives of different neuropeptides families that are assembled may vary depending on the phylogenetic perspective taken.

## NPY/NPF-Type Neuropeptides

**Figure 3:**
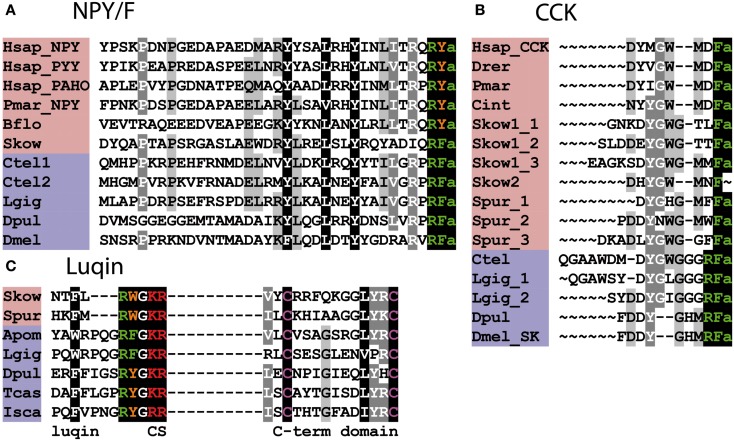
**Alignment of known orthologous peptides from (A) bilaterian NPY/NPF, (B) bilaterian Cholecystokinin/Gastrin (CCK), and (C) bilaterian Luqin/RYamide families**. The names of deuterostomian and protostomian peptides are shaded in pink and blue, respectively. The C-terminal amide groups are represented by an “a” at the end of aligned sequences. Skow Luqin is from Ref. (7), Spur Luqin is from Ref. (75), and Tcas Luqin/RYamide is from Ref. (76). The *Lottia gigantea* sequences are from Ref. (4), the *Capitella teleta* sequences are from Ref. (5), and other sequences are from Ref. (8). For vertebrate sequences, only one peptide from each precursor is shown in the alignment. Genbank (GI) or JGI IDs of all precursor sequences are listed below: **(A)** Alignment of bilaterian NPY/F-type peptides: Hsap_NPY, Hsap_PYY, and Hsap_PAHO; *Homo sapiens* Neuropeptide Y, Peptide Tyrosine tyrosine, and Pancreatic polypeptide precursors (GI:189273, GI:300068955, and GI:35589); Pmar_NPY, *Petromyzon marinus* Neuropeptide Y precursor (GI:57231270); Bflo, *Branchiostoma floridae* NPY-like precursor (GI:260829184); Skow, *Saccoglossus kowalevskii* NPY-like precursor (GI:585716458); Ctel1-2, *Capitella teleta* NPF precursors (GI:161315271, JGI:204022); Lgig, *Lottia gigantea* NPF precursor (GI:163562021); Dpul, *Daphnia pulex* NPF precursor (GI:168841503); Dmel, *Drosophila melanogaster* NPF precursor (GI:442619467). **(B)** Alignment of bilaterian Cholecystokinin/Sulfakinin-type peptides: Hsap, *Homo sapiens* CCK precursor (GI:84040232); Drer, *Danio rerio* CCK-like precursor (GI:42490839); Pmar, *Petromyzon marinus* CCK-like precursor (GI:299891581); Cint, *Ciona intestinalis* CCK-like (Cionin) precursor (GI:296983); Skow1-2, *Saccoglossus kowalevskii* CCK-like precursors (GI:585688033, GI:187061456); Spur, *Strongylocentrotus purpuratus* CCK-like precursor (GI:390355380); Ctel, *Capitella teleta* Sulfakinin (SK)-type precursor (GI:161296032); Lgig, *Lottia gigantean* SK-type precursor (GI:163526260); Dpul, *Daphnia pulex* SK-type precursor (JGI:242979); Dmel, *Drosophila melanogaster* SK-type precursor (GI:386765036). **(C)** Alignment of bilaterian Luqin-type peptides: in addition to the luqin sequences, which are followed by a dibasic cleavage site (CS), the sequences of a conserved cysteine-containing C-terminal domain of the precursor proteins are also shown. The residues connecting the two domains are not represented. Skow, *Saccoglossus kowalevskii* Luqin-like precursor (GI:187205184); Spur, *Strongylocentrotus purpuratus* Luqin-type precursor (GI:390331827); Apom, *Alvinella pompejana* Luqin-type precursor (GI:223786475), Lgig, *Lottia gigantea*, Luqin-type precursor (GI:163510328); Dpul, *Daphnia pulex*, RYamide-type precursor (JGI:251691); Tcas, *Tribolium castaneum*, RYamide-type precursor (GI:347807413); Isca, *Ixodes scapularis* RYamide-type precursor (GI:156462907).

NPY-type neuropeptides include the mammalian peptides neuropeptide Y (NPY), pancreatic polypeptide (PP), and peptide YY (PYY), which have a C-terminal RYamide motif (73). However, some representatives of this family in non-mammalian vertebrates have a C-terminal RFamide motif – e.g., alligator PP (74). An NPY-type neuropeptide in the cephalochordate *B. floridae* also has a C-terminal RYamide motif but the NPY-type neuropeptide in the hemichordate *S. kowalevskii* has a C-terminal RFamide motif (8). Protostomian orthologs of the NPY family are characterized by a C-terminal RFamide motif and hence are named NPF (54) (Figure 3A). Thus, it appears that in the NPY/NPF-type neuropeptide family the C-terminal RYamide motif is a derived characteristic of the chordates and the ancestral motif in the common ancestor of bilaterians was probably RFamide. Also of relevance here are the PrRP-type neuropeptides in mammals and other vertebrates, one of five families of RFamide-type peptides discussed above, which are thought to have arisen by duplication of a gene encoding an NPY/NPF-type precursor in a common ancestor of the deuterostomes (see above). Thus, when taking a bilaterian phylogenetic perspective, the NPY/NPF-type family of neuropeptides can be considered as members of the heterogeneous assemblage of neuropeptides that are categorized as RFamides.

## CCK/Gastrin-Type Neuropeptides

Cholecystokinin (CCK) and gastrin are gut hormones/neuro- peptides in mammals and other vertebrates that share a common C-terminal motif – GWMDFamide (77, 78) (Figure 3B). Furthermore, a CCK/gastrin-type neuropeptide identified in the urochordate *C. intestinalis* also has this motif (79). However, the first invertebrate representatives of the CCK/gastrin family to be identified were the sulfakinins (SKs), which have been isolated from several insect species, and these neuropeptides have a C-terminal RFamide motif (80, 81). In the nematode *C. elegans*, CCK/gastrin-type peptides have a C-terminal QFamide motif (82); however, this is probably a derived feature because CCK/gastrin-type peptides in lophotrochozoan protostomes (e.g., annelids, mollusks) are like the insect SKs in having a C-terminal RFamide motif (4, 5, 8) (Figure 3B). Thus, from a protostomian perspective CCK/gastrin-type neuropeptides are RFamides.

**Figure 4:**
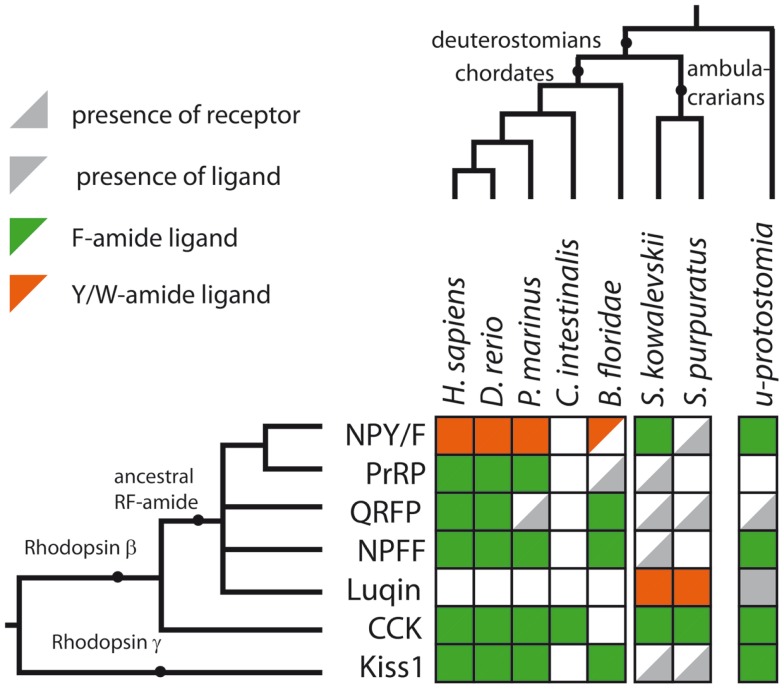
**Summary of RFamide-type peptides and receptors that have been identified in representative deuterostomian taxa**. A gray triangle in the bottom right indicates that a receptor has been identified but a candidate ligand has not yet been identified. A gray triangle in the top left indicates that a peptide(s) has been identified but a candidate cognate receptor has not yet been identified. An empty white square indicates that neither a receptor nor a candidate peptide has been identified. The color of the triangle indicates that the peptide is either a Phe-amide (Fa, green), a Tyr-amide, or Trp-amide (Y/Wa, orange) or unknown (gray) in a given species or group of species. The last column of squares to the right describes the expected situation in the last common ancestral protostomian (*u-protostomia*). A supplementary table provides further details of IDs for the sequence data used to compile this figure.

CCK/gastrin-type neuropeptide precursors have recently been identified in non-chordate deuterostomian invertebrates, providing important new insights on the evolution of this neuropeptide family. In the hemichordate *S. kowalevskii*, there is a CCK/gastrin-type precursor, which gives rise to three putative peptides that have the C-terminal motifs GTLFamide, GTTFamide and GMMFamide [(8); Figure 3B], while in the echinoderm *S. purpuratus*, there is a CCK/gastrin-type precursor that gives rise to three putative peptides that have the C-terminal motifs GMFFamide, GMWFamide and GGFFamide [(8); Figure 3B]. Thus, these ambulacrarian CCK/gastrin-type peptides do not have a C-terminal RFamide motif and therefore, it appears that only the C-terminal Famide motif is a common feature that is found in both protostomian and deuterostomian CCK/gastrin-type peptides (Figures 3B and 4).

## Luqins

Luqin is an RFamide-type neuropeptide (APSWRPQGRFamide) that was originally isolated from the mollusk *Aplysia californica* and named luqin because it is expressed in the dorsal left upper quadrant (LUQ) cells of the abdominal ganglion in this species (83). Subsequently, luqin-type neuropeptides with a C-terminal RFamide motif have been identified in other molluscan species and in annelids (4, 5), while members of the luqin family in arthropods and nematodes are characterized by a C-terminal RYamide motif and hence are referred to as RYamides (8, 76) (Figure 3C). Luqin-type neuropeptides have also been identified in hemichordates and echinoderms and these peptides have a C-terminal RWamide motif (7, 75) (Figure 3C). However, luqin-type neuropeptide signaling has been lost in the chordate lineage (8).

Thus, in conclusion, the luqins are a bilaterian neuropeptide family but only from a lophotrochozoan perspective are they RFamide-type neuropeptides.

## FMRFamide-Related Neuropeptides in Protostomes

Last but not least, we return to where the RFamide story began – with the tetrapeptide FMRFamide that was isolated from molluscan ganglia in 1977 (13). FMRFamide-related peptides that share with FMRFamide a C-terminal FxRFamide motif have been identified throughout the protostomes (15, 16) but not in deuterostomes. Thus, it appears that neuropeptides with the C-terminal FMRFamide-type motif FxRFamide are a uniquely protostomian invention.

## The RFamide Motif: An Ancient and a Convergent Feature of Neuropeptide Evolution

A primary objective here was to review evidence of the occurrence of RFamide-type peptides and their receptors in deuterostomian invertebrates and a summary of our findings is shown in Figure 4 and in a complementary supplementary table. With an anthropocentric perspective, the main focus of this review has been on five types of RFamide neuropeptides that are found in vertebrates: (1) Gonadotropin-inhibitory hormone (GnIH)-type, (2) NPFF-type, (3) Pyroglutamated RFamide peptide (QRFP)-type, (4) Prolactin-releasing peptide (PrRP)-type, and (5) Kisspeptin-type. Four of these neuropeptide types, GnIH, NPFF, QRFP, and PrRP, exert their effects via receptors that belong to distinct clade of the rhodopsin-β-type G protein-coupled neuropeptide–receptors, as illustrated in Figure 1A. Not all of the receptors in this clade are activated by RFamide-type neuropeptides (e.g., tachykinin receptors and leucokinin receptors). But some of the invertebrate members of this receptor clade are activated by RFamide-type peptides (e.g., luqin, NPF), while their vertebrate counterparts have either been lost or are activated by peptides with C-terminal motifs that are structurally similar to RFamide (e.g., RYamide in NPY-type peptides). Therefore, we speculate that the common ancestor of this clade of receptors may have been activated by a neuropeptide with a C-terminal RFamide motif. If this is correct, then we have a scenario where multiple gene duplications followed by diversification has given rise to neuropeptides where the C-terminal RFamide motif has either been retained, slightly modified (e.g., RYamide or RWamide) or lost, but with different patterns of retention, modification, or loss occurring in different branches of the animal kingdom.

But the RFamide motif is not unique to neuropeptide ligands of the clade of rhodopsin-β-type G protein-coupled receptors that includes GnIH-, NPFF-, QRFP-, and PrRP-type receptors. As highlighted above, the ligands for CCK/gastrin-type receptors in protostomian invertebrates also have an RFamide motif (Figures 1A and 3B). This may be a consequence of convergent evolution, with CCK/gastrin-type neuropeptides having acquired an RFamide motif in the protostomian lineage. However, an alternative, and more provocative, hypothesis would be that the RFamide motif in protostomian CCK/gastrin-type neuropeptides reflects conservation of an ancient motif that was a characteristic of the neuropeptide ligand that activated the receptor that is the common ancestor of all rhodopsin-β-type G protein-coupled neuropeptide–receptors in bilaterians.

In this scenario, neuropeptide diversification following multiple gene duplications would have resulted in loss of the RFamide motif in many of the neuropeptides that act as ligands for rhodopsin-β-type G protein-coupled neuropeptide–receptors in bilaterians. Support for the notion of an ancient RFamide neuropeptide signaling system can be found in the discovery that neuropeptides with an RFamide-type motif are present not only in bilaterians but also in basal animal groups such as the cnidarians (7, 84). However, the receptors that mediate the effects of RFamide-type neuropeptides in cnidarians have, as yet, not been identified and it is possible, of course, that receptors for RFamides in cnidarians are not rhodopsin-β-type receptors. Furthermore, the RFamide motif does appear to be a convergent feature of neuropeptide systems and this can be seen in kisspeptins, which exert effects via rhodopsin-γ-type G protein-coupled receptors. Thus, neuropeptides with a C-terminal RFamide motif act as ligands for both rhodopsin-β-type G protein-coupled receptors (GnIH-, NPFF-, QRFP-, and PrRP-type receptors) and rhodopsin-γ-type G protein-coupled receptors (kisspeptin-type receptors).

Interestingly, the abundance of neuropeptide types that share a C-terminal RFamide-type or RFamide-like motif (e.g., RYamide) could, in principle, give rise to cross-talk between neuropeptide signaling systems. Thus, a promiscuous RFamide receptor could potentially bind multiple RFamide-type neuropeptides derived from a variety of different precursor proteins. However, evidence that this occurs physiologically has, to the best of our knowledge, not been obtained. Demonstrating it will require evidence of not only peptide-receptor interaction *in vitro*, but also *in vivo*.

In conclusion, the RFamide motif appears to be both an ancient and a convergent feature of neuropeptide evolution, which then poses the ultimate question as to why this is. It has been proposed that from an enzymatic perspective, the occurrence of an arginine residue in the penultimate position may be a preferred characteristic for cleavage at neighboring monobasic or dibasic cleavage sites. Furthermore, non-polar and aromatic (e.g., phenylalanine and tyrosine) amino acids may be favored substrates for C-terminal amidation (15). Another observation that could explain the occurrence of C-terminal aromatic residues (Phe, Tyr, and Trp) in many neuropeptides, including RFamides, is the fact that rhodopsin α, a class of receptors phylogenetically related to neuropeptide–receptors (85) (see also Figure 1A), bind monoamine neurotransmitters (dopamine, norepinephrine, tyramine, and serotonin), which all have an aromatic functional group that is similar to the aromatic amino acids. This observation raises the possibility that ancestral neuropeptide ligands for rhodopsin-type receptors could have been peptides with a C-terminal aromatic amino acid (Phe/Tyr/Trp-amides).

## Conflict of Interest Statement

The authors declare that the research was conducted in the absence of any commercial or financial relationships that could be construed as a potential conflict of interest.

## Supplementary Material

The Supplementary Material for this article can be found online at http://www.frontiersin.org/Journal/10.3389/fendo.2014.00093/abstract
